# Ischemia modified albumin increase indicating cardiac damage after experimental subarachnoid hemorrhage

**DOI:** 10.1186/1471-2202-15-33

**Published:** 2014-02-24

**Authors:** Şerefden Açıkgöz, Nurullah Edebali, Figen Barut, Murat Can, İshak Özel Tekin, Çağatay Büyükuysal, Bektaş Açıkgöz

**Affiliations:** 1 Department of Biochemistry, Faculty of Medicine, Bülent Ecevit University (Formerly, Zonguldak Karaelmas University), 67630, Esenköy, Kozlu, Zonguldak, Turkey; 2 Department of Neurosurgery, Faculty of Medicine, Bülent Ecevit University (Formerly, Zonguldak Karaelmas University), Zonguldak, Turkey; 3 Department of Pathologia, Faculty of Medicine, Bülent Ecevit University (Formerly, Zonguldak Karaelmas University), Zonguldak, Turkey; 4 Department of Immunologia, Faculty of Medicine, Bülent Ecevit University (Formerly, Zonguldak Karaelmas University), Zonguldak, Turkey; 5 Department of Biostatistics, Faculty of Medicine, Bülent Ecevit University (Formerly, Zonguldak Karaelmas University), Zonguldak, Turkey

**Keywords:** Ischemia modified albumin, Cardiac damage, Experimental subarachnoid hemorrhage

## Abstract

**Background:**

Cardiac complications are often developed after subarachnoid hemorrhage (SAH) and may cause sudden death of the patient. There are reports in the literature addressing ischemia modified albumin (IMA) as an early and useful marker in the diagnosis of ischemic heart events. The aim of this study is to evaluate serum IMA by using the albumin cobalt binding (ACB) test in the first, second, and seventh days of experimental SAH in rats.

Twenty-eight Wistar albino rats were divided into four groups each consisting of seven animals. These were classified as control group, 1st, 2nd and 7th day SAH groups. SAH was done by transclival basilar artery puncture. Blood samples were collected under anesthesia from the left ventricles of the heart using the cardiac puncture method for IMA measurement. Histopathological examinations were performed on the heart and lung tissues. Albumin with by colorimetric, creatine kinase (CK), aspartate aminotransferase (AST), lactate dehydrogenase (LDH) were determined on an automatic analyser using the enzymatic method. IMA using by ACB test was detected with spectrophotometer.

**Results:**

Serum IMA (p = 0.044) in seventh day of SAH were higher compared to the control group. Total injury scores of heart and lung tissue, also myocytolysis at day 7 were significantly higher than control group (p = 0.001, p = 0.001, p = 0.001), day 1 (p = 0.001, p = 0.001, p = 0.001) and day 2 (p = 0.001, p = 0.007, p = 0.001). A positive correlation between IMA - myocytolysis (r = 0.48, p = 0.008), and between IMA – heart tissue total injury score (r = 0.41, p = 0.029) was found.

**Conclusion:**

The results revealed that increased serum IMA may be related to myocardial stress after SAH.

## Background

Cardiac effects of intracranial hemorrhage were initially described in 1903 by Cushing, who noted alterations in blood pressure and cardiac rhythm in patients [1].

There are three main theories explaining the pathogenesis of subarachnoid hemorrhage (SAH)-induced cardiac dysfunction [2]. The first is a multi-vessel coronary artery spasm causing ischemia. Microvascular dysfunction was identified as an another factor. Catecholamine hypothesis was the most responsible [2]. The catecholamine mediated injury is the likely cause of cardiac damage [3-5]. Cathecolamine secretion, even in toxic amounts, from sympathetic nerve terminals within the myocardium have been reported [6].

In literature, cardiac complications, including cardiac arrhytmias [7,8], wall motion abnormalities [7,9], left ventricular dysfunction [10,11], myocardial necrosis [1,4], pulmonary edema [12-14] were noted in SAH patients and experimental SAH models.

The dramatic end of the afore mentioned abnormalities was the sudden death of the patients.

In diagnosis, electrocardiographic changes [7-9,11,15,16] and biochemical cardiac markers such as creatine kinase (CK), CKMB [4,5,9,15,17], and cardiac troponin (cTn) [1,4,7,15] have been used.

Since in cardiac events the increases in serum amounts of intracellular enzymes and proteins occur after tissue necrosis [18], those mentioned markers may not be sensitive in periods before necrosis or in cases where necrosis was not found.

The N terminal of albumin molecule was the primary site where transitional metals such as Co(II), Cu(II) and Ni(II) bind [19]. During acute ischemic conditions, the metal binding capacity of albumin for transition metals is reduced to what is commonly known as ischemia modified albumin (IMA) [20]. IMA may be potentially used as an early marker of myocardial ischemia before necrosis occurs [19,21,22]. Serum IMA was measured using the albumin cobalt binding (ACB) test that measures the binding capacity of albumin to cobalt [19]. It has been approved by the US Food and Drug Administration [23].

In this study the role of IMA, as an early marker, in myocardial stress after experimental SAH was investigated. In rats, after experimental SAH, the changes in IMA on the 1st, 2nd and 7th days were noted and compared with control group animals. Also histopathologic changes in heart and pulmonary tissues were investigated.

## Methods

The study protocol was approved by the Bülent Ecevit University (Formerly, Zonguldak Karaelmas University), and the Animal Ethics commitee (Ethics approval number: B.30.2.Z.K.Ü.0.20.00.00/HADYEK-39).

### Subjects

The animal study was performed at the Experimental Surgery, Animal Care and Research Unit of Bulent Ecevit University, Faculty of Medicine, Zonguldak. Twenty-eight male adult Wistar albino rats, weight 200–300 g, were included in the study. All rats were kept at standard conditions, 21 ± 2°C, appropriate humidity, a 12 h-12 h light–dark cycle, and given sufficient fluids and food. The rats were divided into four groups as follows: first day group (n = 7), second day group (n = 7), seventh day group (n = 7) with SAH and control group (n = 7).

### Surgical procedure

Rats were anesthetized by intraperitoneal injection of ketamine (60 mg/kg) and xylazine (10 mg/kg). Experimental SAH was created with a technique similar to that described by Barry et al. [24]. Briefly, through a midline cervical incision in the supine position, using an operating microscope (Takagi OM-5, Japan) the anterior parapharyngeal approach was used to expose the clivus. A bony window in front of the basilar artery was created using large bore needles with extreme care not to open the prepontine cistern. A suture needle with an outer diameter of 75 μm (Ethicon, Scotland, UK) was inserted into the basilar artery. Withdrawal of the needle caused an extensive hemorrhage into the subarachnoid space with an even distribution up to the olfactory area.

The rats were kept alive for the 1st, 2nd, and 7th days, after SAH under appropriate conditions.

Under anesthesia, blood samples of the animals were collected using the cardiac puncture method from left ventricles then the heart and lung tissues of the animals were removed immediately for histopathologic evaluation.

### Laboratory tests

After the blood clotted, the samples were centrifuged and the serum was immediately stored at −80°C until assayed for analysis. Serum albumin levels (g/dL) using the colorimetry method (Bromocresol Green [BCG]), (intra-assay CV of < %1.1, inter-assay CV of < %1.8, analytical range 1-6 g/dL), creatine phosphokinase (CK U/L) (intra-assay CV of < %1.9, inter-assay CV of < %4.6, analytical range 0-1300U/L), aspartate aminotransferase (AST U/L) (intra-assay CV of < %2.5, inter-assay CV of < %3.3, analytical range 0-1000U/L), and lactate dehydrogenase (LDH U/L) (intra-assay CV of < %0.9, inter-assay CV of < %1.3, analytical range 20-700U/L) activity using the enzymatic method were assayed with Advia 2400 unit (Siemens, Tarrytown, USA).

The levels of serum IMA was measured by the ACB test using Bar- Or’s method [19]. The purpose of the test was the colorimetric measurement of coloured complex of cobalt, unconjugated to serum albumin after cobalt-albumin binding has occured, with ditiyotreitol (DTT). Using a spectrophotometer (Shimadzu UV‒1601, Tokyo, Japan) at 470 nm, color development with DTT was compared to a serum-cobalt blank without DTT and noted as absorbance unit (ABSU) (Intra-assay CV of < %3.5 and inter-assay CV of < %6.1, analytical range 0.324-0.897 ABSU).

### Histopathologic evaluation

Tissue specimens from the lung and heart were fixed in 10% neutral formalin solution and embedded in paraffin. Sections were cut with a cryostat at 4–5 μm thickness from the paraffin blocks of each tissue. Specimens were then deparaffinized and stained with hematoxylin and eosin (H&E). A pathologist microscopically examined the H&E stained sections in blinded fashion, recording results for each the lung and the heart tissue injury. A light microscope was used for evaluation. Tissue damage severity was semi-quantitatively assessed. Scores were given as absent (0), slight change (1), moderate change (2), and severe change (3) for each criteria. The injury criteria used for each tissue for the scoring systems is described below:

Lung: 1) interstitial edema, 2) hemorrhage, 3) airway epithelial-cell damage, 4) hyaline membrane formation, 5) neutrophil infiltration, 6) emphysematous changes, 7) lymphocytic infiltration and 8) total lung injury score. Heart: 1) myocardial edema, 2) myocytolysis, 3) focal hemorrhage, 4) leukocytic infiltration, 5) loss of cross striation, and 6) total lung injury score. The microscopic score of each tissue was calculated as the sum of the scores given to each criteria.

### Statistical analysis

The Statistical Package for Social Sciences 13.0 (SPSS Inc, Chicago, IL, USA) was used in all data analyses. The results were expressed as median, minimum and maximum. The normality distribution of the variables was tested using the Shapiro Wilk test. For the variables without normal distribution, the Kruskal Wallis test was used for the four group comparisons. The Mann Whitney *U*-test with the Bonferroni adjustment was used for two group comparisons of significant variables among the four groups. Spearman’s correlation analysis was used to determine the relationship between continuous variables state measurement. p-values less than 0.05 were considered statistically significant.

## Results

### Biochemical findings

Seventh day group showed significantly enhanced *Median ± (Min-Max)* serum IMA values 0,798 (0,495 – 0,897) absorbance units (ABSU) compared with control group 0,452 (0,347-0,614) ABSU (p = 0.044). Serum albumin, CK, AST, and LDH levels of the 1st, 2nd, and 7th day groups did not have significant differences compared to control group (p = 0.991, p = 0.077, p = 0,646, p = 0,178) respectively (Table 1).

**Table 1:** Levels of LDH, AST, CK and IMA in the serum of SAH and normal rats

	**Control group**	**Day 1 group**	**Day 2 group**	**Day 7 group**	**p value**
	**n = 7**	**n = 7**	**n = 7**	**n = 7**	
IMA(ABSU)	0,452 (0,347 - 0,614)	0,553 (0,341 – 0,864)	0,700 (0,324 – 0,857)	0,798 (0,495 – 0,897)*	0.044
Albumin (g/dL)	3,4 (3,1 – 4,0)	3,6 (3,1 – 3,8)	3,6 (3,1 – 3,9)	3,6 (3,3 – 3,7)	0.991
CK (U/L)	859 (532–2492)	533 (417–2508)	760 (638–2649)	1134 (340–11024)	0.077
AST (U/L)	145 (99–178)	244 (118–315)	151 (114–392)	223 (109–597)	0.646
LDH (U/L)	635 (446–856)	618 (213–3054)	1042 (469–5521)	1222 (447–3559)	0.178

Median (Min - Max).

Significant difference (p < 0,05); *different from control group.

A positive correlation between the IMA - myocytolysis (r = 0.48, p = 0.008) and between IMA–heart tissue total injury score (r = 0.41, p = 0.029) were given in Table 2. There were not significant correlation between IMA and LDH (r = 0.103, p = 0.601), AST(r = 0.199, p = 0.309) and CK(r = 0.269, p = 0.166) (Table 2).

**Table 2:** Correlation coefficients of IMA and heart, lung tissues total injury scores

**(n =28)**	**Correlation coefficient (r)**	**p value**
IMA-myocytolysis	0.480	0.008
IMA-heart total injury score	0.410	0.029
IMA-lung total injury score	0.250	0.191
IMA-LDH	0.103	0.601
IMA-AST	0.199	0.309
IMA-CK	0.269	0.166

Significant difference (p < 0,05).

### Histopathologic findings

The histopathologic examination findings of lung and heart tissues at days 1, 2 and 7 compared with normals were given in Tables 3 and 4. Total injury scores of heart tissues (p = 0.001, p = 0.001, p = 0.001), lung tissues (p = 0.001, p = 0.001, p = 0.001) and myocytolysis (p = 0.004, p = 0.001, p = 0.001) at days 1, 2 and 7 were found significantly higher when compared with the control group. Total injury scores of heart and lung tissue, also myocytolysis at day 7 were significantly higher than day 1 (p = 0.001, p = 0.001, p = 0.001) and day 2 (p = 0.001, p = 0.007, p = 0.001).

**Table 3:** Median histopathologic scores for the lung and heart tissues from each group

	**Control group**	**Day 1 group**	**Day 2 group**	**Day 7 group**	**p value**
	**n = 7**	**n = 7**	**n = 7**	**n = 7**	
**Lung**					
Interstitial edema	0 (0 – 1)	1 (1 – 2)	1 (1 – 2)	2 (2 – 3)	<0,001
Hemorrhage	1 (0 – 1)	2 (2 – 2)	2 (2 – 2)	3 (2 – 3)	<0,001
Epithelial injury	0 (0 – 1)	1 (1 – 2)	1 (1 – 2)	2 (1 – 2)	<0,001
Hyalin membrane formation	0 (0 – 0)	1 (1 – 1)	1 (1 – 2)	2 (1 – 2)	<0,001
Neutrophil infiltration	0 (0 – 1)	2 (1 – 2)	2 (1 – 2)	2 (2 – 3)	<0,001
Emphysematous changes	0 (0 – 1)	1 (1 – 2)	2 (1 – 2)	2 (2 – 3)	<0,001
Lymphocytic infiltration	1 (0 – 1)	1 (1 – 2)	2 (1 – 2)	2 (1 – 2)	0,017
Total injury score	2 (1 – 4)	9 (8 – 13)	11 (9 – 13)	15 (11 – 18)	<0,001
**Heart**					
Myocardial edema	0 (0 – 0)	1 (1 – 2)	1 (1 – 2)	2 (2 – 2)	<0,001
Myocytolysis	0 (0 – 1)	1 (1 – 2)	2 (1 – 2)	3 (3 – 3)	<0,001
Focal hemorrhage	0 (0 – 1)	1 (1 – 2)	2 (1 – 2)	3 (3 – 3)	<0,001
Leukocytic infiltration	0 (0 – 0)	1 (0 – 2)	1 (1 – 2)	2 (1 – 2)	<0,001
Loss of cross striation	0 (0 – 0)	1 (1 – 1)	1 (1 – 1)	2 (1 – 2)	<0,001
Total injury score	0 (0 – 2)	5 (4 – 9)	7 (6 – 8)	11 (10 – 12)	<0,001

Median (Min - Max).

**Table 4:** Inter groups significance in the lung and heart tissues scores

	**p value**					
	**Control – Day1**	**Control – Day2**	**Control – Day7**	**Day1– Day2**	**Day1– Day7**	**Day2– Day7**
**Lung**						
Interstitial edema	0,004	0,002	0,001	0,710	0,002	0,011
Hemorrhage	0,001	0,001	0,001	1,000	0,260	0,026
Epithelial injury	0,004	0,002	0,001	0,710	0,260	0,073
Hyalin membrane formation	0,001	0,001	0,001	0,209	0,260	0,383
Neutrophil infiltration	0,001	0,001	0,001	1,000	0,128	0,128
Emphysematous changes	0,007	0,001	0,001	0,209	0,170	0,128
Lymphocytic infiltration	0,038	0,017	0,017	0,710	0,710	1,000
Total injury score	0,001	0,001	0,001	0,209	0,001	0,007
**Heart**						
Myocardial edema	0,001	0,001	0,001	0,710	0,004	0,026
Myocytolysis	0,004	0,001	0,001	0,073	0,001	0,001
Focal hemorrhage	0,026	0,001	0,001	0,073	0,001	0,001
Leukocytic infiltration	0,004	0,001	0,001	0,456	0,128	0,383
Loss of cross striation	0,001	0,001	0,001	1,000	0,026	0,260
Total injury score	0,001	0,001	0,001	0,026	0,001	0,001

Significant difference (p < 0,0083).

## Discussion

Cardiac complications after SAH may cause sudden death [14,25]. There are studies in the literature adressing the cardiac problems seen after SAH. The authors mostly identified increased sympathetic activity.

In dogs, experimental studies revealed high plasma catecholamine levels [4,5,9], myocardial necrosis [4], micro infarctions with electron microscopy [9], left ventricular dysfunction [5,11] and wall motion abnormalities [9].

In clinical studies, in SAH patients, chances of ECG, high cardiac markers [1,7,15,25], wall motion abnormalities [7,25] were noted. Szabo et al. investigated myocardial perfusion with myocardial scintigraphy. They reported that myocardial ischemia might be seen without specific ECG changes [26].

In their post mortem examinations, Doshi & Neil-Dwyer found necrosis of individual muscle fibers, increased eosinophilia and granular cytoplasm, necrotic muscle fibers surrounded by macrophages [3]. Sugiuro et al., in a case report (50 year old women), noted ECG changes indicating myocardial damage, high cardiac enzymes, hypokinetic left vetricular septum, pulmonary edema. Autopsy of the patient revealed diffuse myocytolysis with coagulation necrosis of the heart muscle without the evidence of coronary artery occlusion [14]. Yuki et al. in their case report reported ECG findings resembling myocardial infarction in a patient. Left ventriculography revealed cardiac dysfunction. Two months later, in the postmortem examination of the patient, after her death from cancer, no evidence of myocardial necrosis was revealed [27]. The authors discussed coronary vasospasm and reversible postischemic stunned myocardium without necrosis which might happened after SAH [27].

In ischemic cardiac events, since intracellular enzymes and proteins increase in serum after the occurence of tissue necrosis [18], they may be insufficient in the diagnosis in the lack of necrosis or in cases where necrosis did not happen yet. Sinha et al., examined IMA, ECG and cTnT in patients who were admitted to an emergency department with acute chest pain. The sensitivity (82%), specifity (46%), negative predictive value (59%), positive predictive value (72%) of IMA were calculated. On contrary the sensitivity of ECG (45%) and cTnT (20%) were noted. They underlined the possible role of IMA in detecting ischemia before necrosis happen [28]. In our experimental SAH study, in rats, we could not demonstrate significant changes in CK, AST and LDH tests when compared with control group at days 1, 2 and 7. There were significant increases of IMA at 7th day after SAH compared with the control group. We observed that the serum albumin values for all groups did not differ from the control group, thus we can say that high IMA in SAH may not be due to low albumin levels.

In the light microscopic examination myocardial necrosis was not seen in all groups but total injury scores of heart and lung tissues at days 1, 2 and 7 were found increased when compared to the control group. Also in days 1, 2 and 7 myocytolysis, which was described by Turillazzi as a specific histological marker of congestive heart failure without relation to coronary blood flow, myocardial hypoxia and myocardial fibrosis [29], was found increased compared to the control group. Both total injury scores of heart and lung tissues and myocytolysis of heart tissue at days 7 were found significantly increased compared to days 1 and 2.

In literature research we did not find an experimental study investigating IMA after experimental SAH.

There were clinical studies in the literature referring to increases of IMA after myocardial ischemia [19], transient myocardial ischemia [21], acute coronary syndrome [22,28].

There were studies investigating IMA changes after pulmonary emboli [30], skeletal muscle ischemia [31-33] and cerebrovascular events including SAH [23,34].

Zapica-Muniz et al., after skeletal muscle forearm ischemia test, in their volunteer subjects, found decreased levels of IMA at 1st, 3rd and 5th minutes compared to baseline values, in addition to low ammonia and high lactate levels. In lactate serum pool the authors found decreased levels of IMA after increases in lactic acid concentrations. The authors underlined the decreased diagnostic sensitivity of IMA because of increased lactate levels [31].

Roy et al., in their patients with peripheral vascular disease (PVD) and leg claudication, performed an exercise test inducing leg ischemia and found decreased levels of IMA during stress peak. They also performed a dobutamin stress echocardiography and found unchanged levels of IMA. In their patients regional wall motion abnormalities were not detected. They noted ischemia of tissues other than myocardium might be responsible for decreases of IMA [32].

IMA levels were found increased in volunteers of a marathon race for 24–48 hours [33]. In another study Falkensammer et al. noted increased IMA levels after calf muscle ischemia induced by exercise in volunteers [35].

Sbarouni et al., in their review article, stated that the changes of IMA after exercises were not homogenous, they might be due to ischemic areas in the gastrointestinal tract and skeletal muscle. They also underlined that the role of hemoconcentration seen after physical exercises, might cause increases of albumin serum levels and because of decreased unbound cobalt to albumin, IMA might be lowered. They noted interference of lactic acidosis with IMA analysis [36].

Han et al. demonstrated increases in IMA after cerebral infarction, intracerebral hemorrhage and SAH (18 patients) compared to control group and they stated a positive correlation between IMA and lipid levels. In this study, patients with pulmonary emboli and coronary artery thrombosis within 6 months were excluded [34].

Gündüz et al. examined and compared the levels of IMA in intracerebral hemorrhage, SAH and brain infarction patients within 24 hours after their symptoms started. They found increased levels of IMA compared to control patients, and in brain infarction the increases were significant compared to SAH, and they stated that this finding might be used in differential diagnosis of SAH with brain infarction. The authors excluded patients with acute coronary syndrome, acute myocardial infarction, cardiac insufficiency, and pulmonary embolism in their study [23].

## Conclusion

In our study we observed increased levels of IMA at days 1 and 2 after SAH, in blood samples obtained by cardiac pucture (left ventricle). We also observed significantly higher levels of IMA at day 7. There was a positive correlation between the IMA and cardiac total injury scores and the cardiac myocytolysis. Our study is an isolated study compared to clinical studies. In the light of our findings we can state that the increases of IMA might be related to a probable myocardial stress after SAH. We believe there is the need for futher studies to find and to develope specific markers besides IMA to detect cardiac stress which may cause the sudden death of patients after SAH.

## Competing interests

The authors declare that they have no competing interests.

## Authors’ contributions

SA conceived the study, in the design of the study, collected and analyzed data, carried out biochemical tests, drafted the manuscript, interpreted data; NE participated collected and analyzed data; carried out in vivo experiments; FB, collected and analyzed data, participated in the carried out histopathology tests; MC, participated in the carried out biochemical tests; IOT participated in the design of the study, collected data; CB participated in the carried out statistical analysis; BA participated in the design of the study, carried out in vivo experiments, interpreted data and coordination of the study. Moreover, all the authors have read and approved the final manuscript.
